# Supplementary material for: Auto QSAR-based active learning docking for hit identification of potential inhibitors of Plasmodium falciparum Hsp90 as antimalarial agents
Source: PLoS One. 2024 Nov 25;19(11):e0308969. doi: 10.1371/journal.pone.0308969 (PMC11588265; doi:10.1371/journal.pone.0308969)
Supplement: S1 File — (DOCX) [file pone.0308969.s001.docx]

Auto QSAR-based Active learning docking for hit identification of potential inhibitors of *Plasmodium falciparum* Hsp90 as antimalarial agents.

Thato Matlhodi1, Lisema Patrick Makatsela 1, Tendamudzimu Harmfree Dongola2, Mthokozisi Blessing Cedric Simelane 3, Addmore Shonhai2, Njabulo Joyfull Gumede4 and Fortunate Mokoena1*

1. Department of Biochemistry, Faculty of Natural and Agricultural Science, North-West University, Mmabatho, South Africa.

2. Department of Biochemistry & Microbiology, University of Venda, Thohoyandou, South Africa.

3. Department of Biochemistry, Faculty of Science, University of Johannesburg, Johannesburg 2006, South Africa.

4. Department of Chemical and Physical Sciences, Faculty of Natural Sciences, Walter Sisulu University (WSU), Private Bag X01, Umthatha, 4099, Eastern Cape, South Africa.

Contact person: Fortunate Mokoena, Fortunate.Mokoena@nwu.ac.za

Department of Biochemistry, North-West University, 1 Albert Luthuli & Cnr University Road, Private Bag X 2046, Mmabatho, 2790, South Africa

# **Supplementary Data**

1. **Reaction based enumeration**

**A**

**B**


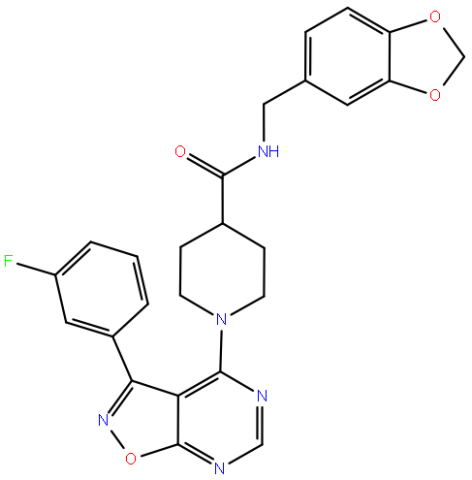

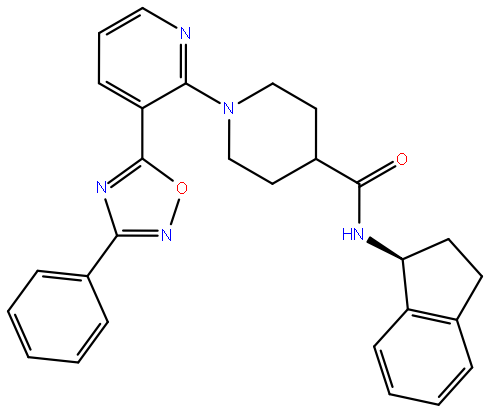


**Figure S1**: reference compounds from the Everson et al 2021 study. A being compound 7 and B being compound 10

**Table S1**:Pathfinder generated reaction pathways -Schrödinger Suite 2019-1

| Path 1 | Amide_coupling-1 |
| --- | --- |
| Path 2 | Amide_coupling-2 |
| Path 3 | Amination-1 |
| Path 4 | Hiyama-1 |
| Path 5 | Negishi |
| Path 6 | Oxadiazole-1 |
| Path 7 | Stille |
| Path 8 | Suzuki |
| Path 9 | Suzuki-2 |

Pathway 6 which is oxadiazole-1 was chosen our pathway of interest shown in figure 1 below

## **AutoQSAR model generation**


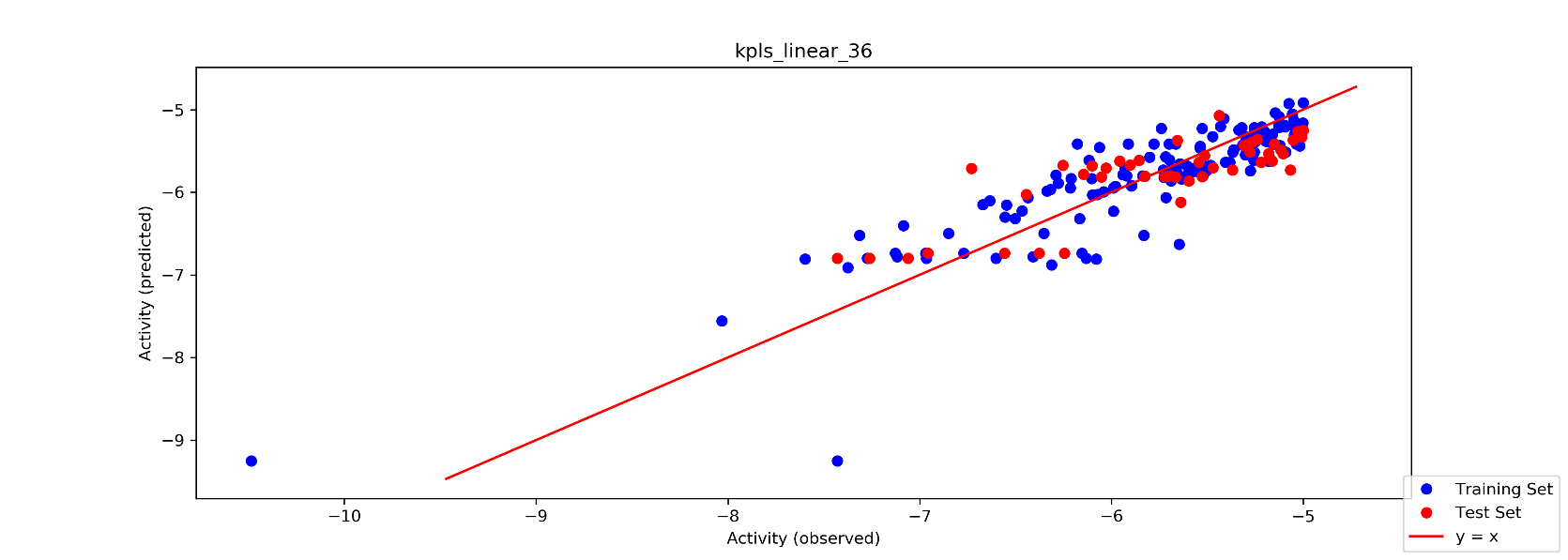


**Figure S2**: model 1


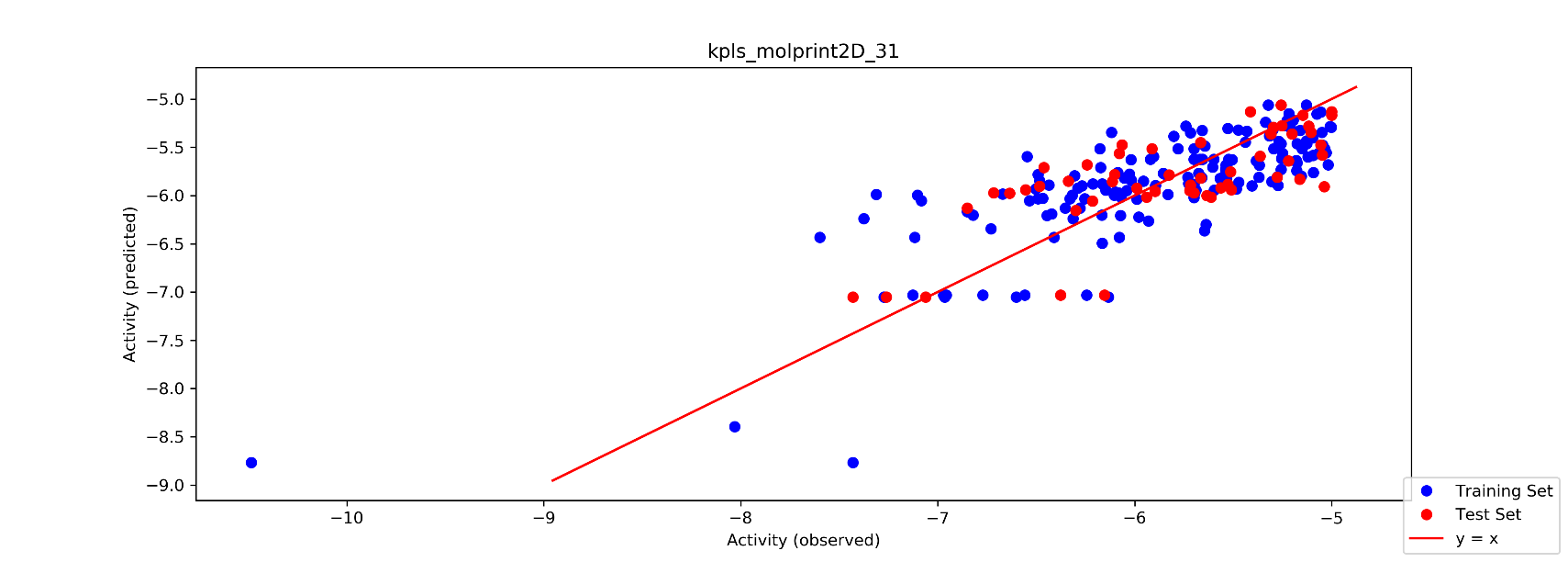


**Figure S3**:model2


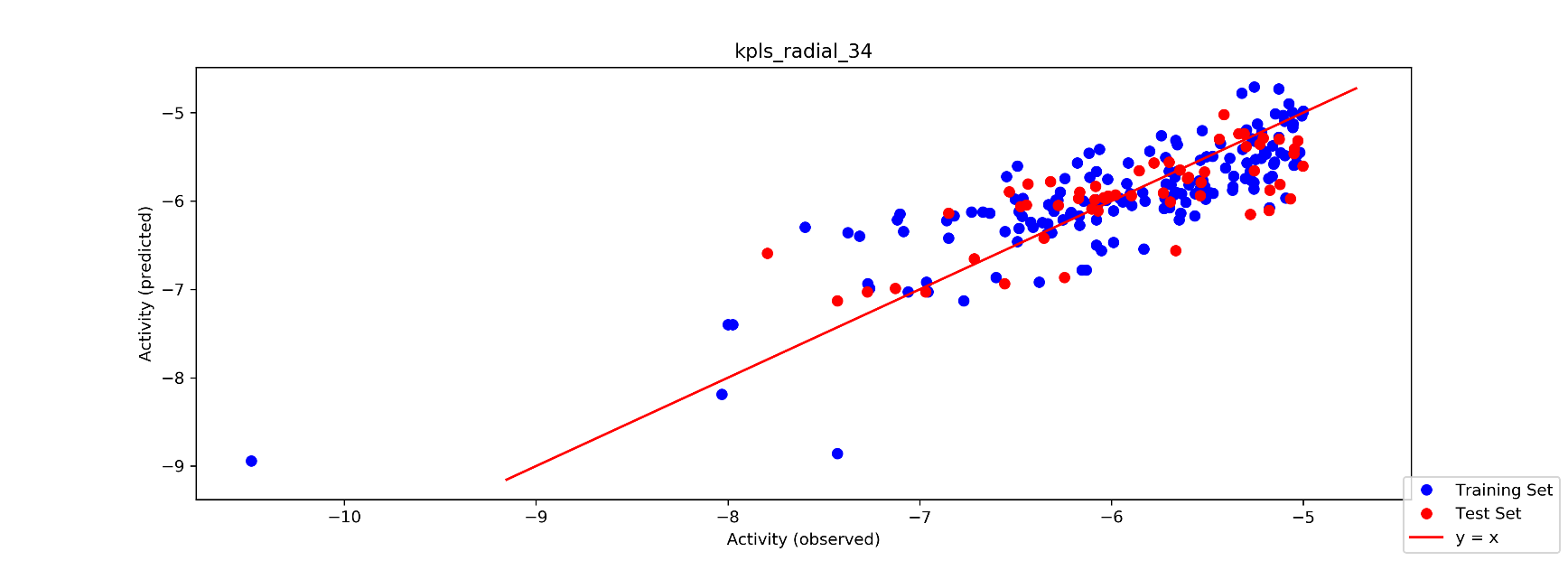


**Figure S4**: model 3


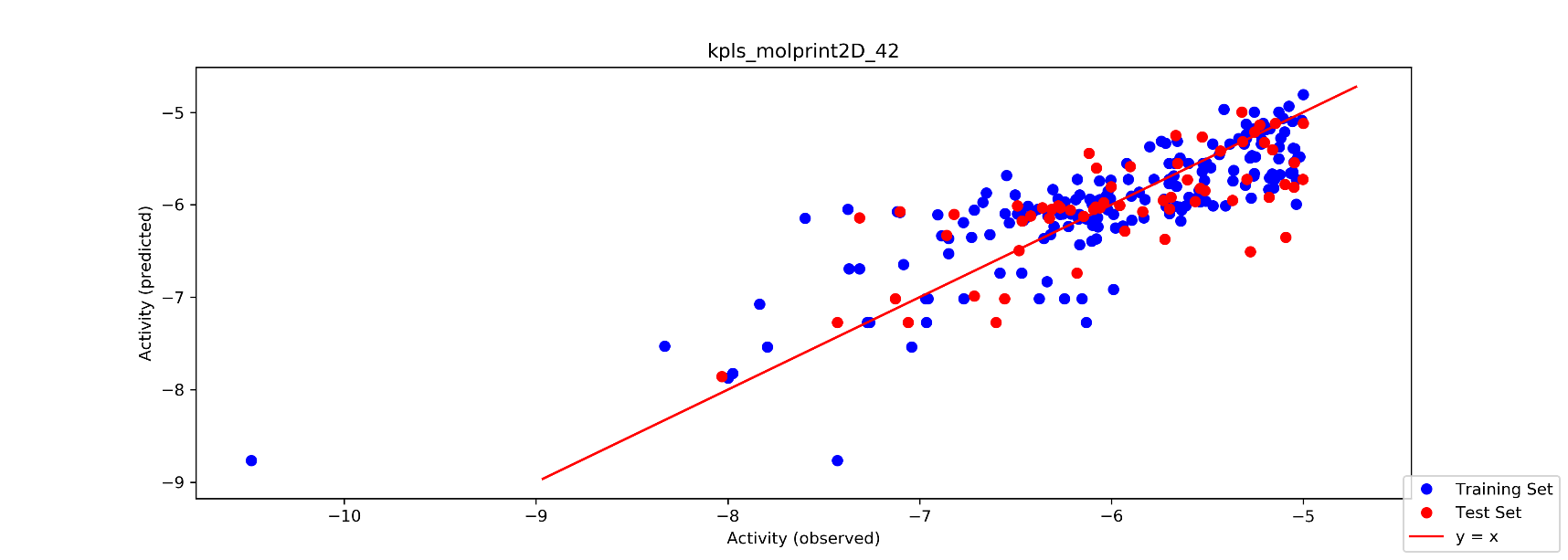


**Figure S5**:model4


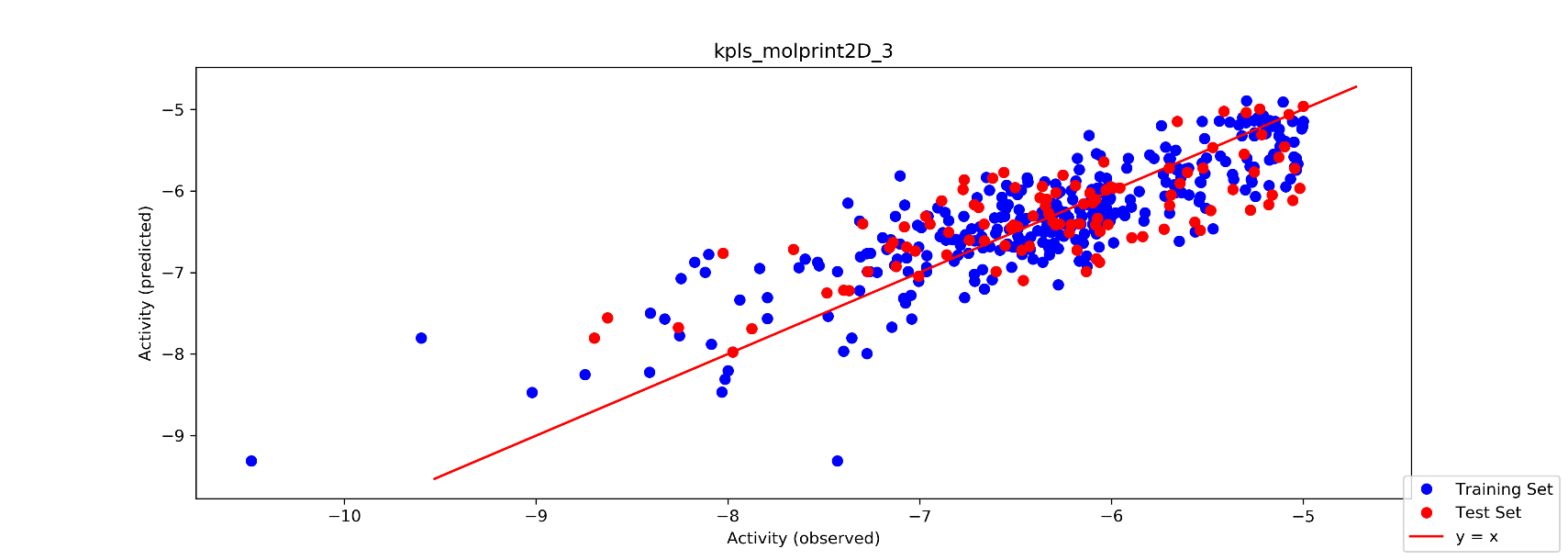


**Figure S6**: model 10

## **Induced fit docking**

**Table S2:** induced fit docking results of the top 62 compounds against the PfHsp90 N-terminal domain receptor

| Title | docking score | glide gscore | glide emodel | XP GScore | IFDScore |
| --- | --- | --- | --- | --- | --- |
| FTN40281232 | -12.032 | -12.058 | -106.600 | -12.058 | -526.97 |
| FTN 2 | -12.351 | -12.351 | -126.680 | -12.351 | -526.86 |
| FTN315699906 | -10.501 | -10.746 | -113.012 | -10.746 | -526.14 |
| FTN315452139 | -10.396 | -10.396 | -117.889 | -10.396 | -525.99 |
| FTN48606212 | -10.796 | -10.796 | -92.753 | -10.796 | -524.35 |
| FTN 3 | -10.380 | -10.457 | -101.975 | -10.457 | -523.50 |
| FTN5 | -11.216 | -11.216 | -103.704 | -11.216 | -523.34 |
| FTN39249872 | -10.178 | -10.431 | -65.285 | -10.431 | -523.26 |
| FTN41168581 | -11.209 | -11.224 | -84.635 | -11.224 | -522.53 |
| FTN313761635 | -10.603 | -10.611 | -86.084 | -10.611 | -520.29 |
| FTN4919 | -13.161 | -13.162 | -108.122 | -13.162 | -533.02 |
| FTN717 | -12.399 | -12.435 | -85.494 | -12.435 | -528.05 |
| FTN1424 | -11.975 | -11.976 | -107.794 | -11.976 | -526.61 |
| FTN1841 | -11.396 | -11.885 | -105.884 | -11.885 | -526.20 |
| FTN1424 | -12.446 | -12.447 | -101.188 | -12.447 | -526.03 |
| FTN 1 | -14.746 | -14.794 | -120.018 | -14.794 | -525.66 |
| FTN3967 | -11.880 | -12.442 | -111.528 | -12.442 | -525.22 |
| FTN4079 | -13.233 | -14.746 | -116.205 | -14.746 | -524.84 |
| FTN1133 | -11.006 | -11.171 | -107.716 | -11.171 | -524.79 |
| FTN 4 | -14.313 | -14.313 | -105.919 | -14.313 | -524.73 |
| FTN1133 | -10.175 | -11.013 | -106.508 | -11.013 | -524.43 |
| FTN1841 | -11.894 | -12.235 | -86.219 | -12.235 | -524.04 |
| FTN1715 | -10.790 | -13.133 | -102.921 | -13.133 | -523.90 |
| FTN3630 | -13.914 | -13.914 | -84.466 | -13.914 | -523.58 |
| FTN1813 | -11.941 | -12.423 | -101.299 | -12.423 | -523.44 |
| FTN4994 | -10.301 | -10.301 | -101.453 | -10.301 | -523.43 |
| FTN3885 | -13.873 | -13.874 | -100.826 | -13.874 | -522.27 |
| FTN4184 | -10.790 | -10.955 | -102.957 | -10.955 | -522.18 |
| FTN4363 | -12.400 | -12.400 | -90.507 | -12.400 | -522.02 |
| FTN3782 | -10.461 | -10.461 | -103.342 | -10.461 | -522.00 |
| FTN1261 | -12.330 | -12.332 | -98.280 | -12.332 | -521.62 |
| FTN3885 | -12.396 | -12.397 | -105.667 | -12.397 | -521.51 |
| FTN499 | -12.397 | -12.759 | -89.158 | -12.759 | -521.49 |
| FTN4363 | -12.281 | -12.281 | -92.724 | -12.281 | -521.47 |
| FTN1715 | -13.770 | -13.781 | -112.874 | -13.781 | -521.33 |
| FTN4843 | -11.544 | -11.544 | -85.764 | -11.544 | -521.27 |
| FTN499 | -11.470 | -11.933 | -115.374 | -11.933 | -521.21 |
| FTN3885 | -11.725 | -11.726 | -105.179 | -11.726 | -521.15 |
| FTN3885 | -11.995 | -11.996 | -108.696 | -11.996 | -521.07 |
| FTN2075 | -11.412 | -11.983 | -117.573 | -11.983 | -520.93 |
| FTN2925 | -10.489 | -11.022 | -119.488 | -11.022 | -520.92 |
| FTN3057 | -11.430 | -11.430 | -78.680 | -11.430 | -520.64 |
| FTN2701 | -10.676 | -10.774 | -104.290 | -10.774 | -520.06 |
| FTN4031 | -10.535 | -10.535 | -91.366 | -10.535 | -519.80 |
| FTN2474 | -12.242 | -12.242 | -89.123 | -12.242 | -519.66 |
| FTN0 | -11.056 | -11.056 | -77.271 | -11.056 | -519.33 |
| FTN2110 | -12.134 | -12.153 | -76.051 | -12.153 | -519.33 |
| FTN1589 | -10.151 | -10.151 | -79.481 | -10.151 | -518.91 |
| FTN2211 | -10.254 | -10.264 | -79.071 | -10.264 | -518.87 |
| FTN1388 | -10.186 | -10.188 | -73.541 | -10.188 | -517.80 |
| FTN1454 | -10.579 | -11.025 | -80.285 | -11.025 | -517.77 |
| FTN3550 | -11.025 | -11.025 | -52.640 | -11.025 | -517.74 |
| FTN3550 | -11.156 | -11.156 | -75.256 | -11.156 | -517.20 |
| FTN3967 | -11.988 | -12.278 | -89.206 | -12.278 | -516.97 |
| FTN3885 | -14.593 | -14.594 | -96.499 | -14.594 | -516.89 |
| FTN3550 | -10.190 | -10.190 | -54.918 | -10.190 | -516.65 |
| FTN1956 | -12.433 | -12.433 | -56.229 | -12.433 | -516.10 |
| FTN4541 | -10.567 | -10.567 | -82.188 | -10.567 | -515.48 |
| FTN2403 | -14.162 | -14.162 | -100.693 | -14.162 | -515.14 |
| FTN3816 | -12.238 | -12.238 | -71.732 | -12.238 | -514.82 |
| FTN95 | -12.502 | -12.502 | -88.717 | -12.502 | -514.19 |
| FTN 6 | -14.176 | -14.176 | -77.866 | -14.176 | -512.26 |

**Human Hsp90 IFD results**

**Table S3** IFD Results of top 62 docked ligands against human Hsp90

| Title | docking score | glide gscore | glide emodel | XP GScore | IFDScore |
| --- | --- | --- | --- | --- | --- |
| FTN 315452139 | -12.108 | -12.108 | -107.792 | -12.108 | -483.60 |
| FTN 4919 | -8.588 | -8.589 | -74.147 | -8.589 | -483.16 |
| FTN 717 | -11.344 | -11.381 | -69.257 | -11.381 | -481.77 |
| FTN 4843 | -8.858 | -8.858 | -69.833 | -8.858 | -480.61 |
| FTN 4994 | -11.274 | -11.274 | -94.124 | -11.274 | -481.07 |
| FTN 40281232 | -10.106 | -10.132 | -119.206 | -10.132 | -480.67 |
| FTN 3967 | -9.843 | -10.405 | -88.841 | -10.405 | -480.67 |
| FTN 1841 | -10.009 | -10.498 | -81.878 | -10.498 | -480.48 |
| FTN 1424 | -7.406 | -7.407 | -88.461 | -7.407 | -480.47 |
| FTN 1424 | -8.161 | -8.162 | -73.416 | -8.162 | -480.20 |
| FTN 3967 | -10.750 | -11.040 | -70.525 | -11.040 | -479.85 |
| FTN 499 | -11.241 | -11.705 | -73.851 | -11.705 | -478.94 |
| FTN 1 | -9.696 | -11.208 | -83.033 | -11.208 | -479.05 |
| FTN 3782 | -9.887 | -9.887 | -93.335 | -9.887 | -479.01 |
| FTN 4079 | -10.721 | -10.769 | -84.547 | -10.769 | -478.69 |
| FTN 39249872 | -9.112 | -9.365 | -101.781 | -9.365 | -478.89 |
| FTN 315699906 | -8.438 | -8.683 | -110.313 | -8.683 | -478.87 |
| FTN 4184 | -9.628 | -9.793 | -78.937 | -9.793 | -478.49 |
| FTN 48606212 | -8.840 | -8.840 | -96.472 | -8.840 | -478.70 |
| FTN 1813 | -9.635 | -10.117 | -86.519 | -10.117 | -478.67 |
| FTN 1133 | -9.546 | -9.711 | -76.544 | -9.711 | -477.99 |
| FTN 1133 | -7.736 | -8.573 | -88.841 | -8.573 | -477.97 |
| FTN 3885 | -11.935 | -11.936 | -88.061 | -11.936 | -477.97 |
| FTN 3885 | -12.258 | -12.259 | -90.306 | -12.259 | -477.85 |
| FTN 4363 | -10.540 | -10.540 | -70.817 | -10.540 | -477.73 |
| FTN 1841 | -9.820 | -10.162 | -61.125 | -10.162 | -477.44 |
| FTN 499 | -9.933 | -10.295 | -69.552 | -10.295 | -477.60 |
| FTN 1715 | -10.042 | -10.054 | -80.589 | -10.054 | -477.40 |
| FTN 1715 | -7.067 | -9.410 | -64.766 | -9.410 | -476.95 |
| FTN 2 | -7.718 | -7.718 | -91.858 | -7.718 | -477.49 |
| FTN 3 | -8.091 | -8.168 | -69.566 | -8.168 | -476.92 |
| FTN 1454 | -7.794 | -8.240 | -60.846 | -8.240 | -477.18 |
| FTN 3885 | -11.247 | -11.248 | -81.836 | -11.248 | -477.02 |
| FTN 313761635 | -9.566 | -9.574 | -91.269 | -9.574 | -476.93 |
| FTN 4 | -10.573 | -10.573 | -80.879 | -10.573 | -476.88 |
| FTN 0 | -10.606 | -10.606 | -73.063 | -10.606 | -476.82 |
| FTN 41168581 | -9.639 | -9.653 | -97.019 | -9.653 | -476.80 |
| FTN 5 | -9.291 | -9.291 | -100.868 | -9.291 | -476.79 |
| FTN 3885 | -11.205 | -11.206 | -85.156 | -11.206 | -476.64 |
| FTN 3885 | -11.356 | -11.357 | -82.290 | -11.357 | -476.22 |
| FTN 4363 | -9.320 | -9.320 | -71.521 | -9.320 | -476.12 |
| FTN 2701 | -9.197 | -9.295 | -58.078 | -9.295 | -475.78 |
| FTN 2075 | -8.427 | -8.997 | -88.691 | -8.997 | -475.72 |
| FTN 2925 | -7.917 | -8.450 | -74.441 | -8.450 | -474.97 |
| FTN 4031 | -8.559 | -8.559 | -67.429 | -8.559 | -475.17 |
| FTN 4541 | -7.923 | -7.923 | -64.967 | -7.923 | -475.04 |
| FTN 3630 | -9.246 | -9.246 | -66.496 | -9.246 | -474.60 |
| FTN 6 | -9.476 | -9.476 | -64.259 | -9.476 | -474.34 |
| FTN 1956 | -8.851 | -8.851 | -70.780 | -8.851 | -474.27 |
| FTN 2474 | -8.353 | -8.353 | -63.745 | -8.353 | -474.23 |
| FTN 1261 | -8.915 | -8.917 | -80.463 | -8.917 | -474.18 |
| FTN 3057 | -8.137 | -8.137 | -54.889 | -8.137 | -474.07 |
| FTN 2110 | -8.201 | -8.220 | -62.872 | -8.220 | -473.94 |
| FTN 2211 | -8.205 | -8.215 | -50.809 | -8.215 | -472.91 |
| FTN 1589 | -7.292 | -7.292 | -57.357 | -7.292 | -473.34 |
| FTN 1388 | -7.042 | -7.044 | -43.262 | -7.044 | -473.23 |
| FTN 3550 | -9.021 | -9.021 | -64.583 | -9.021 | -473.18 |
| FTN 2403 | -8.844 | -8.844 | -61.754 | -8.844 | -472.83 |
| FTN 3816 | -8.956 | -8.956 | -54.034 | -8.956 | -472.53 |
| FTN 3550 | -8.071 | -8.071 | -57.252 | -8.071 | -472.15 |
| FTN 3550 | -8.064 | -8.064 | -54.193 | -8.064 | -471.96 |
| FTN 95 | -8.160 | -8.160 | -57.789 | -8.160 | -470.98 |


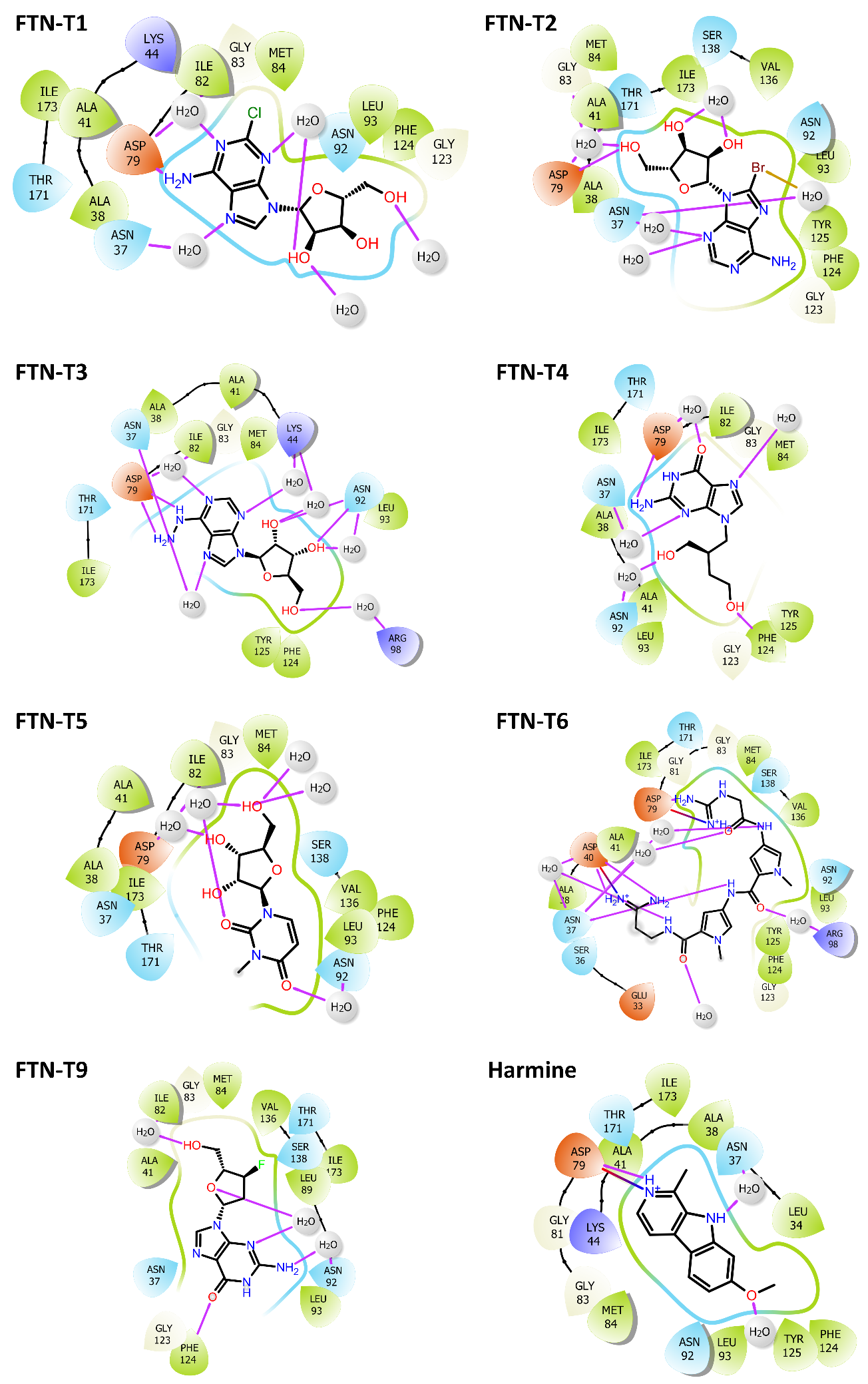


**Figure S7**:2D ligand interaction diagrams for the Induced fit docking of compounds FTN-T1, FTN-T2, FTN-T3, FTN-T4, FTN-T5, FTN-T6, FTN-T9 and Harmine against PfHsp90.

**
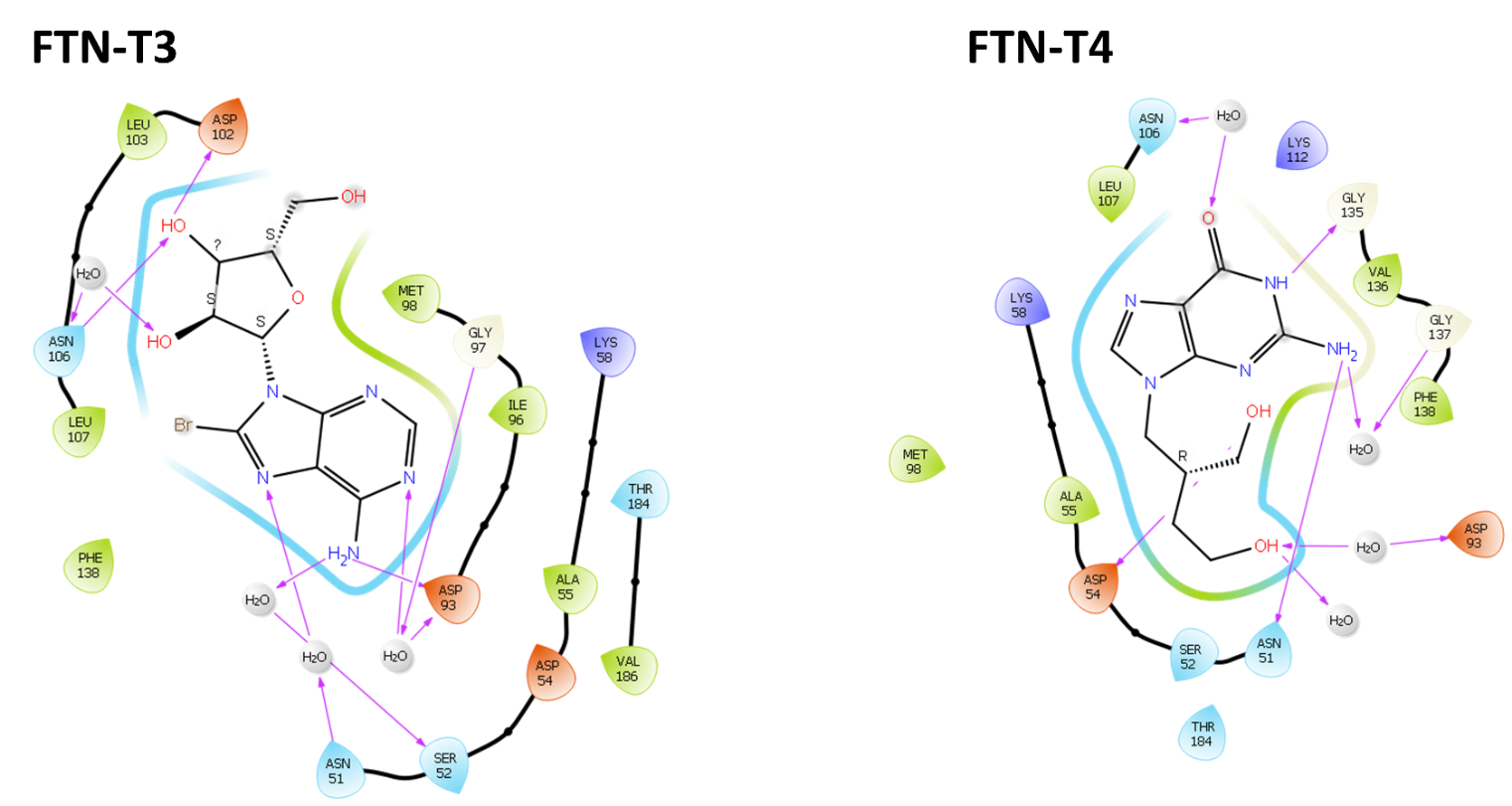

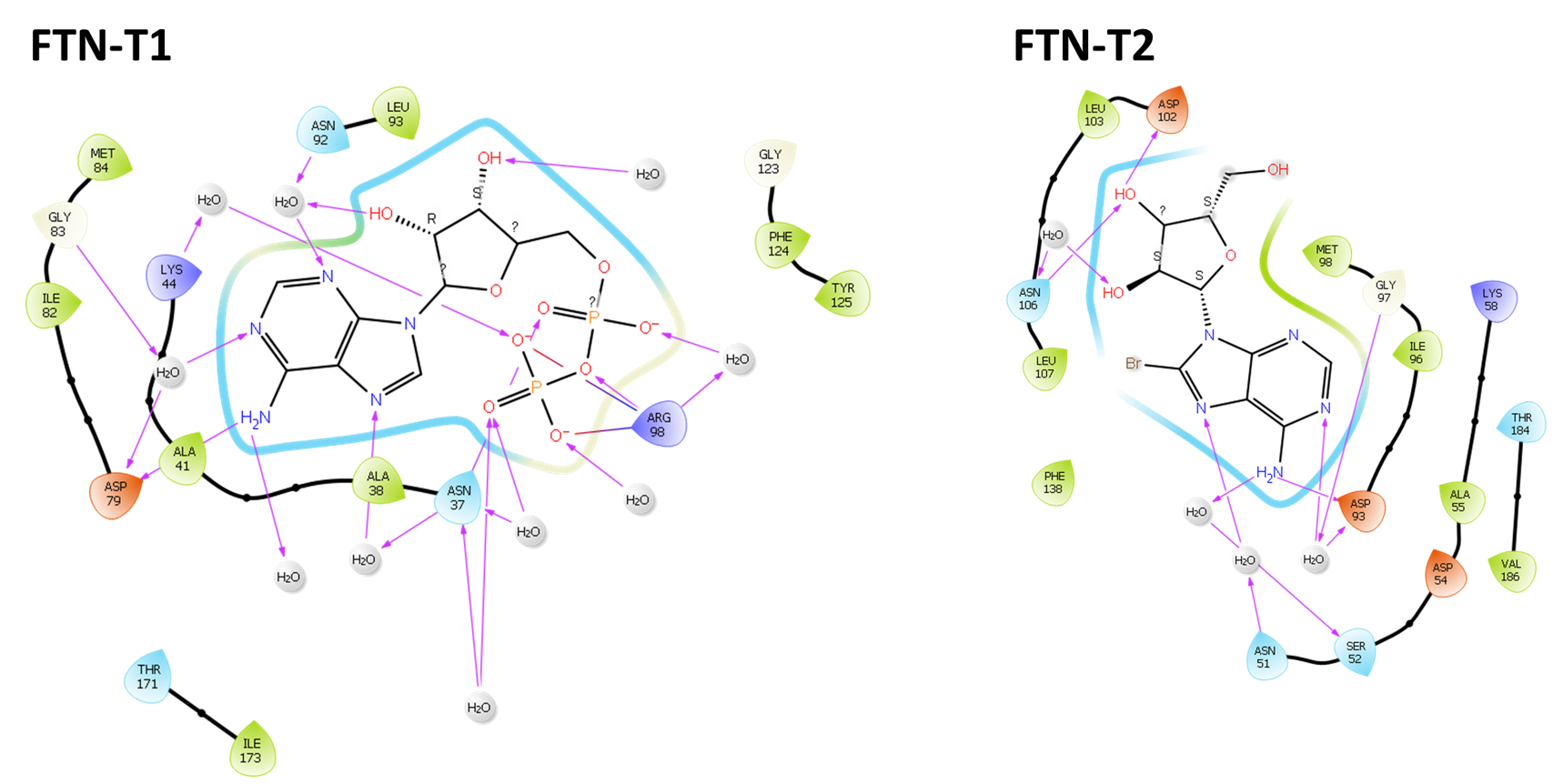
**

**
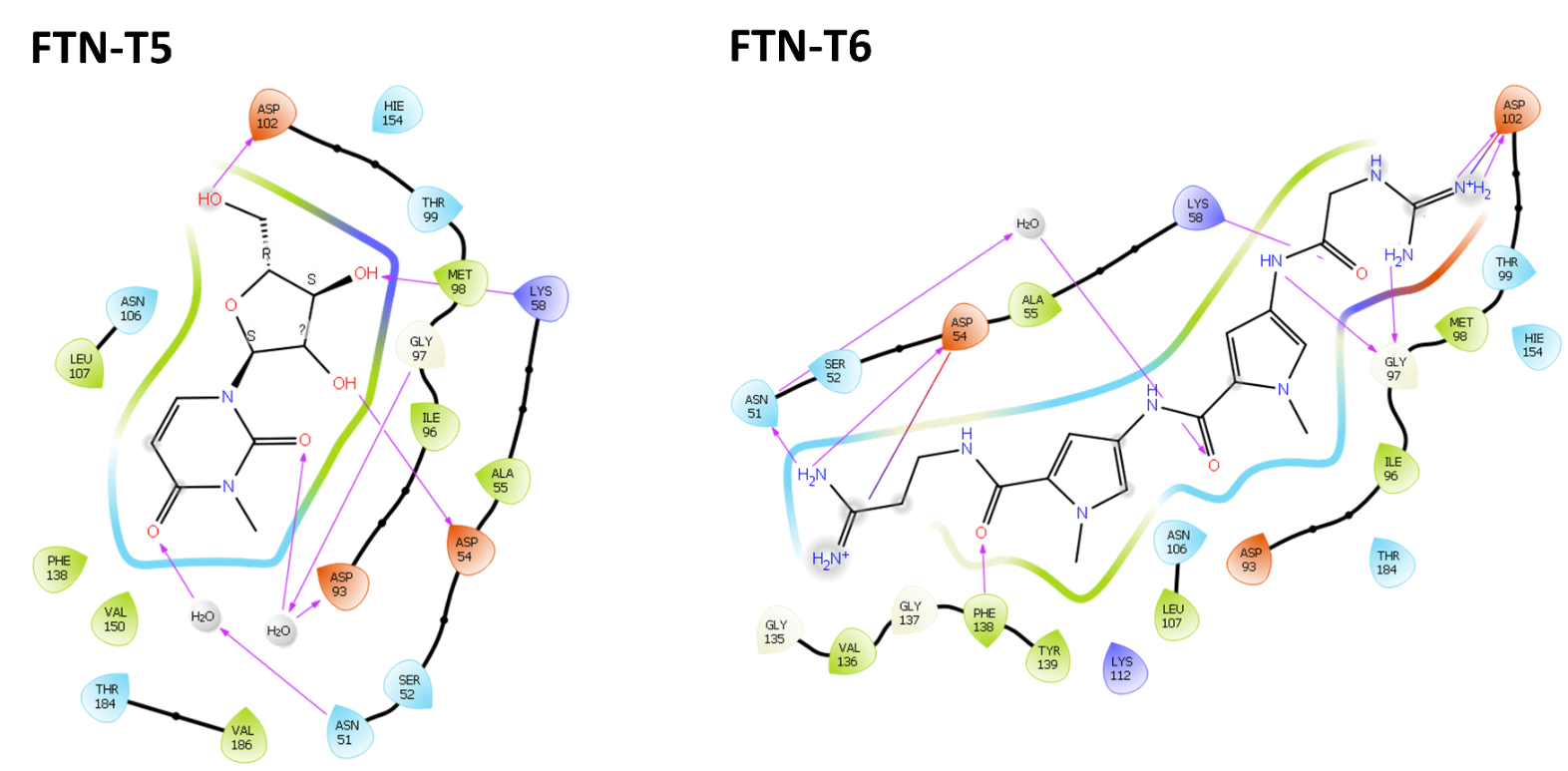
**

**
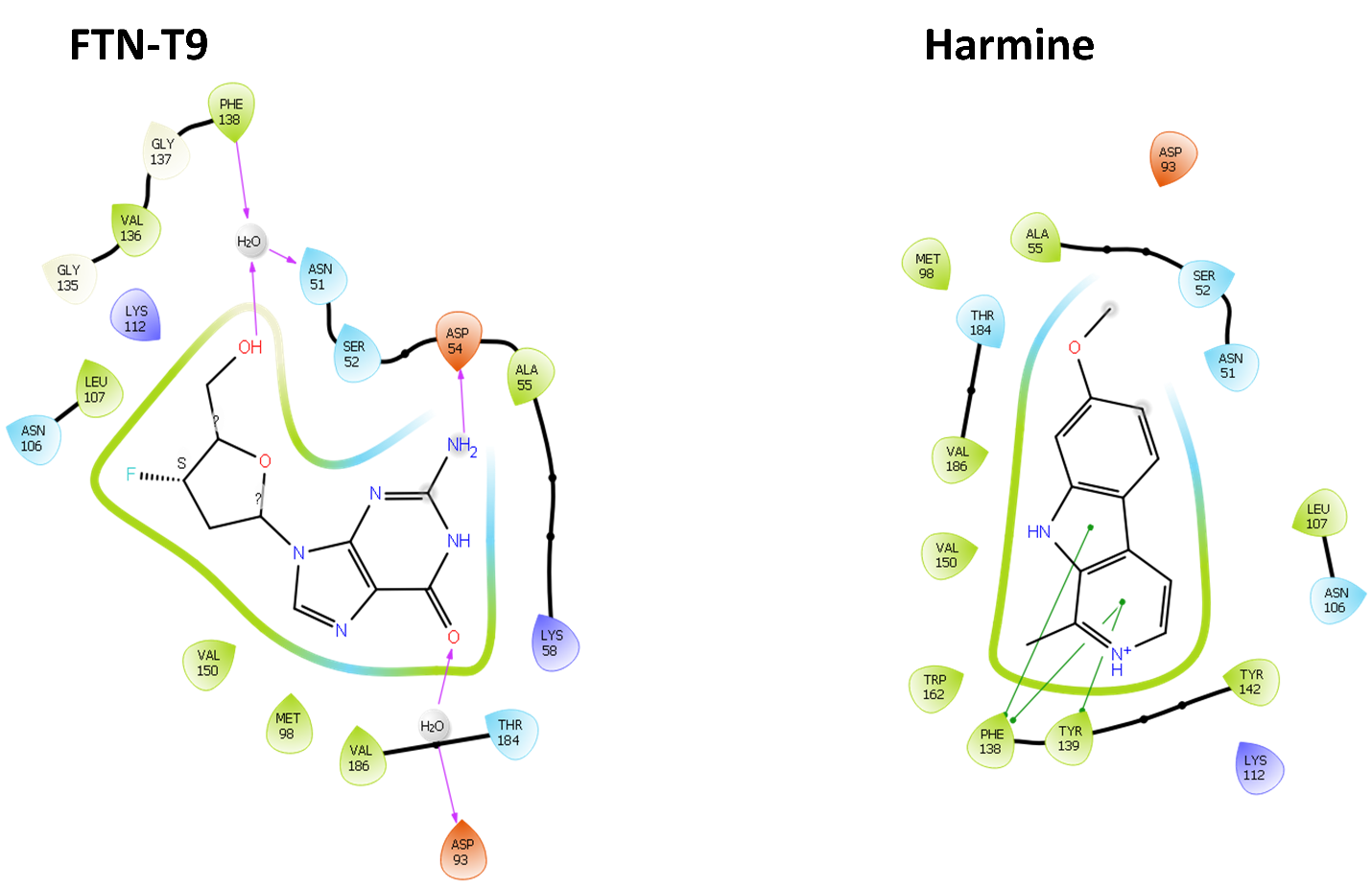
**

**Figure S8**: 2D ligand interaction diagrams for the Induced fit docking of compounds FTN-T1, FTN-T2, FTN-T3, FTN-T4, FTN-T5, FTN-T6, FTN-T9 and Harmine against Human Hsp90.

## **ADME/T properties**

Table S5: ADME/T properties of the top 62 compounds

| Title | mol MW | QPpolrz | QPlog  Po/w | QPlogS | QPlog  HERG | QPPCaco | QPlog  BB | QPlog  Kp | HOA | PSA | Rule  Of  5 | Rule  Of  3 |
| --- | --- | --- | --- | --- | --- | --- | --- | --- | --- | --- | --- | --- |
| 40281232 | 592.696 | 66.296 | 2.890 | -7.322 | -5.272 | 12.603 | -1.499 | -4.338 | 1 | 131.590 | 1 | 3 |
| 37475529 | 593.724 | 63.129 | 5.441 | -6.796 | -5.423 | 513.824 | -1.197 | -1.431 | 1 | 115.289 | 2 | 2 |
| 315699906 | 583.692 | 66.102 | 3.077 | -6.024 | -6.025 | 44.643 | -1.646 | -4.057 | 2 | 152.695 | 2 | 2 |
| 315452139 | 593.644 | 64.297 | 3.195 | -6.684 | -5.667 | 44.406 | -2.321 | -3.953 | 1 | 188.602 | 2 | 2 |
| 48606212 | 583.643 | 60.676 | 4.420 | -6.184 | -3.901 | 19.856 | -2.182 | -2.932 | 2 | 154.053 | 2 | 3 |
| 265681303 | 552.678 | 63.066 | 3.812 | -6.170 | -6.688 | 52.291 | -1.322 | -4.514 | 1 | 134.183 | 1 | 2 |
| 314876700 | 594.712 | 65.812 | 4.556 | -6.867 | -6.839 | 132.426 | -0.989 | -3.722 | 1 | 121.105 | 1 | 2 |
| 39249872 | 517.630 | 58.734 | 2.308 | -4.557 | -5.158 | 19.826 | -1.651 | -4.984 | 2 | 144.343 | 1 | 2 |
| 41168581 | 582.701 | 64.243 | 3.291 | -6.046 | -7.054 | 31.507 | -1.946 | -4.667 | 2 | 139.510 | 1 | 2 |
| 313761635 | 552.678 | 61.555 | 2.611 | -4.131 | -7.726 | 6.099 | -1.445 | -6.775 | 1 | 142.656 | 2 | 2 |
| 4919 | 458.450 | 42.540 | -1.603 | -1.992 | -1.797 | 0.068 | -3.557 | -7.504 | 1 | 202.064 | 2 | 1 |
| 717 | 435.391 | 35.055 | -0.181 | -2.476 | -1.285 | 0.716 | -3.243 | -5.854 | 1 | 181.872 | 2 | 1 |
| 1424 | 466.496 | 49.631 | 1.349 | -4.615 | -2.969 | 0.424 | -4.517 | -7.834 | 1 | 211.092 | 2 | 1 |
| 1841 | 377.339 | 34.114 | 1.306 | -2.517 | -1.909 | 2.591 | -2.524 | -4.171 | 1 | 133.084 | 0 | 1 |
| 1424 | 466.496 | 48.971 | 1.408 | -4.553 | -2.789 | 0.734 | -4.170 | -7.490 | 1 | 200.730 | 2 | 1 |
| 4079 | 391.707 | 29.985 | -0.454 | -2.611 | -0.764 | 0.503 | -2.814 | -6.589 | 1 | 175.631 | 2 | 1 |
| 3967 | 302.229 | 22.445 | -0.923 | -1.430 | -0.549 | 0.393 | -3.121 | -6.588 | 1 | 171.012 | 1 | 1 |
| 4079 | 391.707 | 29.471 | -0.426 | -2.472 | -0.516 | 0.593 | -2.644 | -6.462 | 1 | 179.514 | 2 | 1 |
| 1133 | 332.255 | 24.474 | -1.263 | -1.194 | -0.255 | 0.521 | -3.007 | -6.381 | 1 | 176.666 | 1 | 1 |
| 1412 | 355.368 | 30.812 | -0.556 | -2.525 | -4.486 | 53.334 | -1.996 | -4.926 | 2 | 147.843 | 0 | 0 |
| 1133 | 332.255 | 25.150 | -1.186 | -1.231 | -0.265 | 0.541 | -3.002 | -6.341 | 1 | 182.509 | 1 | 1 |
| 1841 | 377.339 | 33.506 | 1.365 | -2.278 | -1.677 | 3.713 | -2.279 | -3.847 | 2 | 134.683 | 0 | 1 |
| 1715 | 346.317 | 26.817 | -2.679 | -1.858 | -4.158 | 4.580 | -2.991 | -7.006 | 1 | 189.347 | 2 | 1 |
| 3630 | 346.140 | 24.016 | -1.375 | -2.072 | -3.782 | 37.891 | -1.786 | -5.464 | 2 | 137.313 | 0 | 0 |
| 1813 | 365.328 | 33.195 | 1.263 | -2.915 | -0.905 | 2.371 | -2.193 | -5.107 | 1 | 134.786 | 0 | 1 |
| 4994 | 445.477 | 46.260 | 1.119 | -5.363 | -6.446 | 13.814 | -3.774 | -7.563 | 2 | 208.195 | 2 | 1 |
| 3885 | 371.343 | 30.645 | -1.081 | -1.971 | -0.927 | 0.279 | -3.501 | -6.761 | 1 | 195.038 | 1 | 2 |
| 4184 | 331.267 | 24.308 | -0.933 | -1.289 | -0.447 | 0.632 | -2.958 | -6.128 | 1 | 161.514 | 0 | 1 |
| 4363 | 255.233 | 20.504 | -1.925 | -1.409 | -3.741 | 16.480 | -2.453 | -5.979 | 2 | 148.929 | 0 | 1 |
| 3782 | 325.280 | 27.637 | -1.265 | -2.106 | -2.824 | 0.750 | -3.630 | -7.322 | 1 | 193.683 | 1 | 1 |
| 1261 | 368.736 | 32.641 | -0.594 | -2.752 | -4.822 | 105.397 | -1.462 | -4.462 | 2 | 138.425 | 1 | 0 |
| 3885 | 371.343 | 30.975 | -1.248 | -2.048 | -1.028 | 0.154 | -3.820 | -7.265 | 1 | 201.425 | 1 | 2 |
| 499 | 337.225 | 25.553 | -2.131 | -1.505 | -0.577 | 0.198 | -3.326 | -7.210 | 1 | 198.069 | 2 | 1 |
| 4363 | 255.233 | 20.135 | -1.952 | -1.396 | -3.844 | 17.910 | -2.450 | -5.894 | 2 | 149.810 | 0 | 1 |
| 1715 | 346.317 | 26.116 | -2.575 | -1.740 | -3.837 | 6.535 | -2.709 | -6.704 | 1 | 186.780 | 2 | 1 |
| 4843 | 273.294 | 26.363 | 0.043 | -2.603 | -4.223 | 53.635 | -1.794 | -5.134 | 2 | 117.908 | 0 | 0 |
| 499 | 337.225 | 24.571 | -2.016 | -1.413 | -0.459 | 0.352 | -3.016 | -6.694 | 1 | 197.388 | 2 | 1 |
| 3885 | 371.343 | 30.820 | -1.181 | -2.030 | -1.032 | 0.202 | -3.697 | -7.031 | 1 | 200.749 | 1 | 2 |
| 3885 | 371.343 | 31.124 | -1.282 | -2.078 | -1.084 | 0.134 | -3.906 | -7.372 | 1 | 202.789 | 1 | 2 |
| 2075 | 401.206 | 29.040 | -1.833 | -1.071 | 0.870 | 0.016 | -4.112 | -7.976 | 1 | 226.764 | 2 | 1 |
| 2925 | 364.092 | 26.700 | -3.021 | -2.143 | -2.039 | 0.172 | -2.358 | -8.496 | 1 | 157.416 | 0 | 1 |
| 3057 | 287.225 | 23.293 | -0.721 | -2.245 | -3.445 | 78.273 | -1.320 | -5.155 | 2 | 124.191 | 0 | 0 |
| 2701 | 422.232 | 27.574 | -2.017 | -0.924 | -0.256 | 0.160 | -3.242 | -7.530 | 1 | 232.169 | 1 | 1 |
| 4031 | 314.346 | 32.370 | -0.233 | -1.903 | -3.395 | 30.380 | -1.952 | -4.867 | 2 | 131.703 | 0 | 0 |
| 2474 | 297.331 | 26.155 | -0.409 | -2.314 | -3.960 | 100.185 | -1.458 | -4.619 | 3 | 115.026 | 0 | 0 |
|  | 337.245 | 26.777 | -0.639 | -3.414 | -3.127 | 0.195 | -4.470 | -8.172 | 1 | 215.135 | 1 | 1 |
| 2110 | 263.252 | 25.052 | -0.622 | -2.135 | -4.342 | 71.307 | -1.655 | -4.745 | 2 | 121.469 | 0 | 0 |
| 1589 | 235.245 | 21.188 | -0.616 | -1.798 | -3.662 | 68.549 | -1.691 | -5.076 | 2 | 119.602 | 0 | 0 |
| 2211 | 259.218 | 24.731 | 1.083 | -2.467 | -1.111 | 1.895 | -2.053 | -5.304 | 1 | 131.089 | 0 | 1 |
| 1388 | 313.206 | 26.665 | -0.970 | -1.211 | -2.057 | 37.289 | -1.111 | -4.296 | 2 | 121.524 | 0 | 0 |
| 1454 | 251.669 | 20.725 | -4.021 | 0.502 | -0.058 | 0.412 | -1.902 | -8.168 | 1 | 160.767 | 1 | 1 |
| 3550 | 268.250 | 23.523 | -0.884 | -0.793 | -4.442 | 40.922 | -0.779 | -6.292 | 2 | 109.229 | 0 | 0 |
| 3550 | 268.250 | 23.705 | -0.951 | -0.937 | -4.685 | 31.777 | -0.926 | -6.525 | 2 | 108.578 | 0 | 0 |
| 3967 | 302.229 | 22.616 | -0.919 | -1.432 | -0.514 | 0.361 | -3.135 | -6.652 | 1 | 169.562 | 1 | 1 |
| 3885 | 371.343 | 29.877 | -1.286 | -1.860 | -0.821 | 0.183 | -3.628 | -7.113 | 1 | 198.727 | 1 | 2 |
| 3550 | 268.250 | 23.734 | -0.959 | -0.995 | -4.670 | 27.745 | -0.963 | -6.666 | 2 | 113.526 | 0 | 0 |
| 1956 | 389.152 | 27.096 | -0.053 | -2.812 | -4.357 | 109.022 | -1.315 | -4.510 | 3 | 117.537 | 0 | 0 |
| 4541 | 221.218 | 20.204 | -1.124 | -1.906 | -3.947 | 42.283 | -1.928 | -5.389 | 2 | 120.798 | 0 | 0 |
| 2403 | 296.285 | 24.650 | -2.190 | -0.598 | -4.456 | 5.770 | -1.731 | -7.801 | 2 | 152.409 | 1 | 1 |
| 3816 | 298.252 | 26.263 | -1.964 | -1.564 | -4.247 | 14.786 | -2.511 | -6.154 | 2 | 167.235 | 0 | 1 |
| 95 | 272.260 | 23.326 | -1.205 | -1.551 | -4.045 | 74.627 | -1.959 | -4.751 | 2 | 134.179 | 0 | 0 |
| 845 | 251.244 | 22.294 | -1.118 | -1.739 | -3.737 | 78.966 | -1.526 | -4.860 | 2 | 119.441 | 0 | 0 |

ation on BL21(DE3) E. coli cells analysis. **(A)** SDS-PAGE stained with Coomassie blue. **(B)** Western blot analysis using His-tagged antibodies. Lane PL represents protein ladder, lane 0(J.C) uninduced cells at time point zero, lane 0 total extract of cells transformed prior to IPTG induction; lane 1 – 5 and 24 are hourly samples and overnight samples respectively, after induction with IPTG; 24(J.C) are uninduced overnight sample. (**C)** SDS-PAGE analysis of PfHsp90 protein purification samples. The purification samples were analysed by SDS-PAGE on a 12 % gel. **(D)** Western blot analysis of the purification samples. M: Molecular mass marker; Lane 2: Filtered lysate (soluble supernatant); Lane 3: Sample flow-through; Lane 4: Wash 1; Lane 5: Wash 2;lane 6: wash 3; Lane 7-9: Elution. lanes

**Figure S12**..

1. **Cross docking results**

**
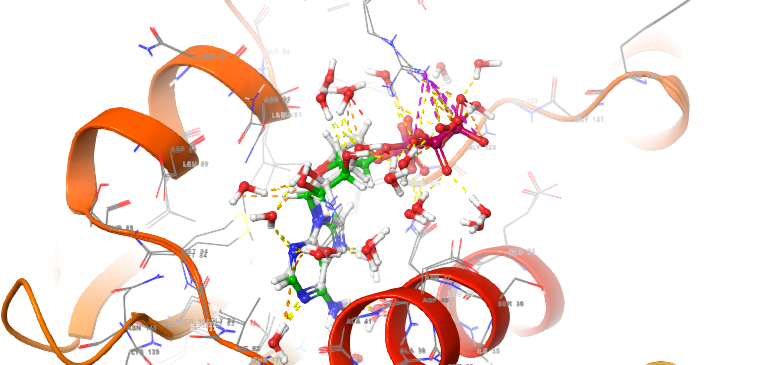

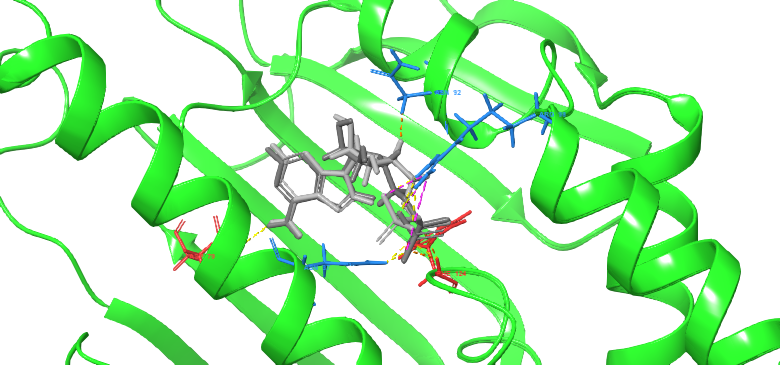
**

**Figure S13:** Cross docking results. To validate the docking procedure, the co-crystalyzed ligand ADP was redocked into the PfHsp90 binding site, which obtained a high degree of overlapping, as seen above with the two superimposed structures perfectly aligning with an RMSD of 0.6565 Å, indicating a reasonably accurate docking procedure.
