# Supplementary material for: Auto QSAR-based active learning docking for hit identification of potential inhibitors of Plasmodium falciparum Hsp90 as antimalarial agents
Source: PLoS One. 2024 Nov 25;19(11):e0308969. doi: 10.1371/journal.pone.0308969 (PMC11588265; doi:10.1371/journal.pone.0308969)
Supplement: Attachment — Submitted filename: 17May2024 Reviewer 2 comments_Finalized.docx [file pone.0308969.s002.docx]

**Auto QSAR-based Active learning docking for hit identification of potential inhibitors of Plasmodium falciparum Hsp90 as antimalarial agents**

**PLOS ONE**

**Reviewer 2 comments**

Matlhod et al used compound 10 as a reference PfHsp90 to generate 10000 models using combinations of computational techniques. These models, together with compounds from the CheMBL data base were subjected to several training and testing rounds to identify compounds with high binding affinity to PfHsp90. A few of the top rank compounds were purchased and evaluated in vitro against the drug susceptible strain of *Plasmodium falciparum*. I will recommend publication of the work subject to the following revision:

(1) **The authors should screen at least the best *in vitro* hit compound from this study against drug resistant strain of Plasmodium falciparum.**

*Response:* *We thank the reviewer for this helpful comment. When we conceptualized the study, we had a screening cascade. The first was to predict the activities of the compounds in silico. Then, promising hits will be purchased, followed by cell and biochemical assays. For the cell-based assay, we thought that to save on cost, only compounds with IC_50_ in the sub-μM range would be progressed for drug-resistant strain testing. This is because current antimalarials exhibit potencies within the single-digit nanomolar range. Therefore, anything lower than 1μM requires extensive optimization before being considered a lead compound.*

(2) **There are several repetitions in the manuscript, especially between the methods and results sections, consider cutting back on these**.
     *Response: Thank you for your constructive feedback regarding the repetitions observed between the methods and results sections of our manuscript. We acknowledge the overlap and appreciate your suggestion to streamline the content for clarity and conciseness. We have carefully reviewed the manuscript and revised it to eliminate redundant information, ensuring that each section distinctly contributes to the overall narrative without unnecessary repetition. Please refer to the track changed versions to keep track of the extensive editing conducted.*
(3) **The manuscript should be sent for thorough language editing as there many incomplete sentences or inappropriate punctuations**.

*Response: Thank you. We hope the revised version of the paper reflects an improvement in punctuations.*

(4) **In both experimental parts, authors have written “Survival was plotted”. This is not very correct, it must be rephrased.**

*Response: Thank you for the feedback. The survival plot shows the number of viable parasites during the dose-dependent determination of IC50, which would determine the concentration of compound required to “kill” 50% of the parasite. Similarly, this was used for the cytotoxicity study. This is standard practice in many anti-malaria drug discovery papers.*

**Since computational methods suggested high binding to humanHsp90, others might consider testing for toxicity against another human cell line like HEK293**

*Response: Yes- this would be a good consideration in the future.*

**Minor comments**

(5) **Page 11, line 62-65, rephrase the sentence ‘’ Furthermore, areas such as Rwanda (4) and East Asia (5) have begun to report the spread and dissemination of first-line treatment options to address artemisinin-tolerant P. falciparum strains to emphasize the urgent need to develop potent and reliable anti-parasitic drugs’’. It is ambiguous.**

*Response: This has been revised to “Countries such as Rwanda (4) and East Asia (5) have begun to report the spread and dissemination of first-line treatment options artemisinin-tolerant P. falciparum strains emphasizing the urgent need to develop potent and reliable anti-parasitic drugs” Please refer to line 71-74 for the revision.*

(6) **Page 11, line 67, reference needed at the end**.

*Response: Thank you for the suggestion to add a reference. This suggestion has been duly noted and the reference added at the end of the sentence in line 77.*

(7) **Page 12, line 75 to 77, references needed at the end of each sentence**.

*Response: Thank you for the comment to include references to this sentence. This suggestion has been noted and the reference added in line 87, page 3 of the manuscript.*

(8) **Page 14, line 131, a figure containing the structure of compound 10 should be included nearby and mentioned in the text.**

*Response: Thank you for your comment regarding the inclusion of a figure containing the structure of compound 10. We appreciate your suggestion and would like to confirm that the figure has been included in the supplementary data as Figure S1 B. Furthermore, we have referenced this figure in the text to ensure its relevance and accessibility to readers*.

(9) **Page 20, line 269, “≥6.5” double check that a minus sign(-) is not missing**

*Response: Thank you for your attention to detail regarding the value "≥6.5" on page 20, line 269 of the manuscript. The necessary correction has been made to accurately represent the value. We appreciate your thorough review and the opportunity to address this concern.*
